# Supplementary material for: Quasi-quantized Hall response in bulk InAs
Source: Sci Rep. 2022 Feb 9;12:2153. doi: 10.1038/s41598-022-05916-2 (PMC8828743; doi:10.1038/s41598-022-05916-2)
Supplement: Supplementary file 1 — Supplementary Information. [file 41598_2022_5916_MOESM1_ESM.pdf]

Quasi-quantized Hall response in bulk InAs

R. Wawrzyńczak[1,*], S. Galeski[1], J. Noky[1], Y. Sun[1], C. Felser[1], and J. Gooth[1,2,]**

[1]Max Planck Institute for Chemical Physics of Solids, 01187 Dresden, Germany
[2]Institut für Festkörper- und Materialphysik, Technische Universität Dresden, 01062 Dresden, Germany
[*]rafal.wawrzynczak@cpfs.mpg.de
[**]johannes.gooth@cpfs.mpg.de

Additional information

Hall conductivity and derivative relation for Sample B.

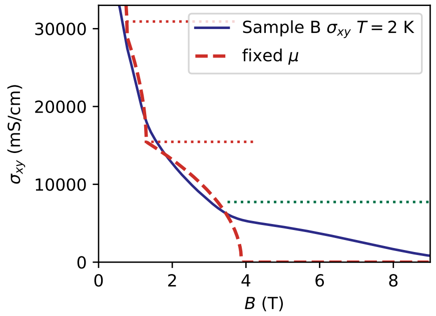

Figure S1. Field dependence of σ_{xy} for sample B. The red, dashed line is the result of fit to the Eq. **??**, which implied the fixed chemical potential of charge carriers. The red, dotted lines mark the values of quasi-quantized Hall conductance for $\nu = 1$ and $\nu = 2$ filling factors. The green dotted line marks the contribution from $\nu = 0^-$ last spin-split Landau level.

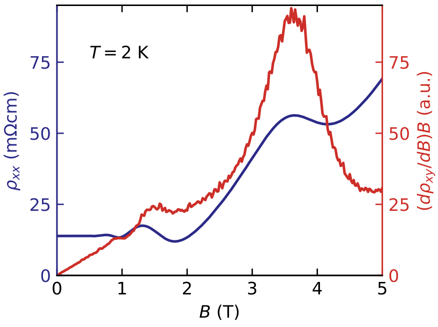

Figure S2. The derivative relation for Sample B. Field dependence of ρ_{xx} is given in absolute units and field dependence of $(d\rho_{xy}/dB)B$ is scaled to fit the ρ_{xx}. I n case of Sample B parameter $\gamma = 0.36$.

Anomalous features in σ_{xy}.

Derivatives with respect to magnetic field strength show the existence of distinct feaures in σ_{xy}, present in the vicinity of fields marking the steps in Hall conductivity calculated for fixed chemical potential in the way described in the main text.

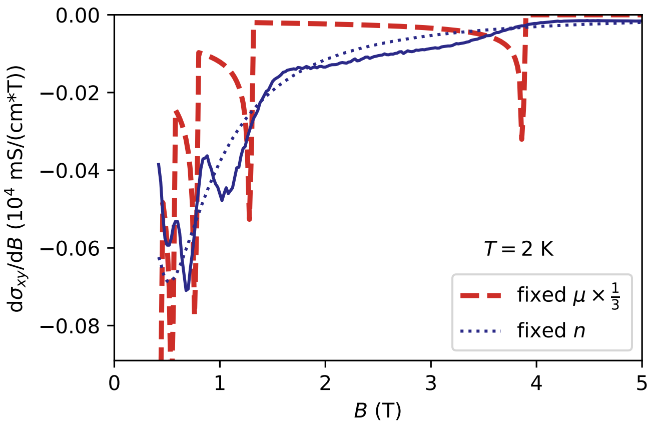

Figure S3. Derivatives with respect to the magnetic field strength of measured and calculated Hall conductivities.

Longitudinal magnetoresistance beyond B_{LLL}.

In Figs. **??**(e-g) showing the oscillatory part of $\rho_{xx}(B)$, apart from the maximum marking the $\mu = 0^+$ spin-split LL one can determine the presence of much smaller shoulder placed in slightly higher fields. Careful examination of ρ_{xx} curves and their derivatives, in the vicinity of B_{LLL} (Fig. **??**), shows that for field directions not parallel to the direction of electric current flow no signs of such a shoulder is distinguishable in the ρ_{xx}. This would suggest the bump at the side of $\nu = 0^+$ peak being an artifact resulting from power-law background subtraction, which does not capture increase in rate change of resistivity in field after crossing B_{LLL}, what could be expected.

On the other hand, ρ_{xx} for $B \parallel x$ does show a bump, easily noticeable thanks to the suppression of $\nu = 0^+$ peak in that configuration. This peak appears in the fields outside of range covered by the theory employed in this work and at this point we cannot account for this feature. We hope that future measurements at sub-Kelvin temperatures, with use of dilution refrigerator, could allow us, by suppressing thermal broadening of energy levels to resolve observed feature in greater detail and address this open question.

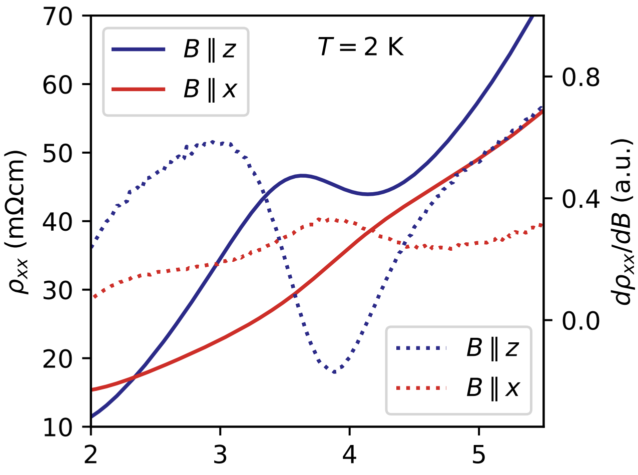

Figure S4. Field dependence of ρ_{xx} and its derivative, around B_{LLL}, for field directions perpendicular and parallel to the electric current.

QQHE in Sample A at low temperatures.

In addition we measure the resitivity of our Sample A at low temperatures using dilution refrigerator for sample A. No significant difference was observed. The only additional feature appearing in Fig. **??** is placed at the value of conductivity corresponding to contribution from three spin-split LLs ($\nu = 0^-$, $\nu = 0^+$ and $\nu = 1^-$). Lower temperatures should be more favorable towards its observation, as thermal smearing of LLs is smaller. However it is hard to confirm its origin with confidence.

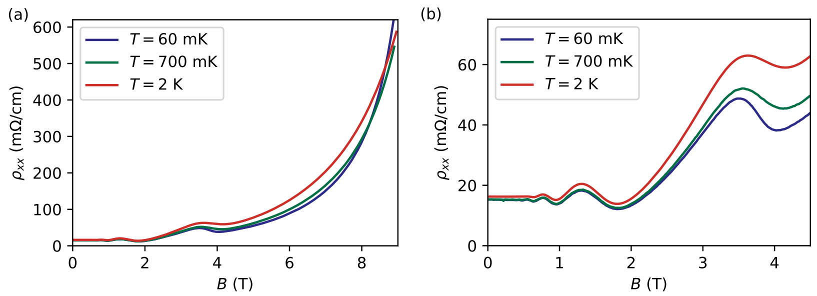

Figure S5. (a) Field dependence of ρ_{xx} at different temperatures. (b) Low-field part of the data, where SdH oscillations can be observed.

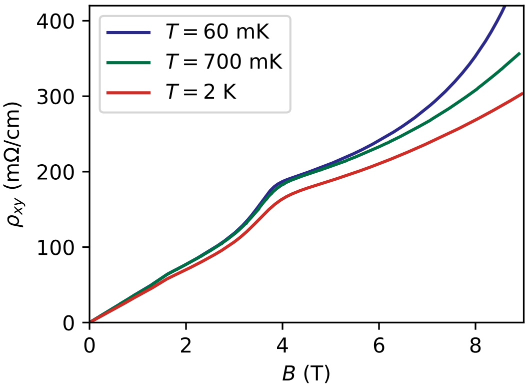

Figure S6. Field dependence of ρ_{xy} at different temperatures.

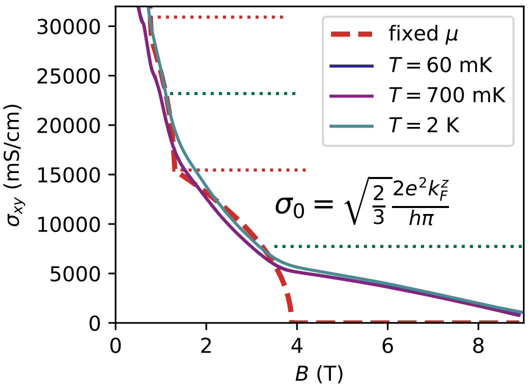

Figure S7. Field dependence of σ_{xy}, the red, dashed line is the result of fit to the Eq. **??** from the main text, which implied the fixed chemical potential of charge carriers. The red, dotted lines mark the values of quasi-quantized Hall conductance for $\nu = 1$ and $\nu = 2$ filling factors. The green dotted lines mark the contribution from $\nu = 0^-$ last spin-split Landau level and three lowest spin split levels, possible contribution of which might be faintly visible in the lowest temperatures.
